# Distinct Temporal Coordination of Spontaneous Population Activity between Basal Forebrain and Auditory Cortex

**DOI:** 10.3389/fncir.2017.00064

**Published:** 2017-09-14

**Authors:** Josue G. Yague, Tomomi Tsunematsu, Shuzo Sakata

**Affiliations:** Strathclyde Institute of Pharmacy and Biomedical Sciences, University of Strathclyde Glasgow, United Kingdom

**Keywords:** acetylcholine, neural ensemble, optogenetics, neural oscillations, neural coding, brain state

## Abstract

The basal forebrain (BF) has long been implicated in attention, learning and memory, and recent studies have established a causal relationship between artificial BF activation and arousal. However, neural ensemble dynamics in the BF still remains unclear. Here, recording neural population activity in the BF and comparing it with simultaneously recorded cortical population under both anesthetized and unanesthetized conditions, we investigate the difference in the structure of spontaneous population activity between the BF and the auditory cortex (AC) in mice. The AC neuronal population show a skewed spike rate distribution, a higher proportion of short (≤80 ms) inter-spike intervals (ISIs) and a rich repertoire of rhythmic firing across frequencies. Although the distribution of spontaneous firing rate in the BF is also skewed, a proportion of short ISIs can be explained by a Poisson model at short time scales (≤20 ms) and spike count correlations are lower compared to AC cells, with optogenetically identified cholinergic cell pairs showing exceptionally higher correlations. Furthermore, a smaller fraction of BF neurons shows spike-field entrainment across frequencies: a subset of BF neurons fire rhythmically at slow (≤6 Hz) frequencies, with varied phase preferences to ongoing field potentials, in contrast to a consistent phase preference of AC populations. Firing of these slow rhythmic BF cells is correlated to a greater degree than other rhythmic BF cell pairs. Overall, the fundamental difference in the structure of population activity between the AC and BF is their temporal coordination, in particular their operational timescales. These results suggest that BF neurons slowly modulate downstream populations whereas cortical circuits transmit signals on multiple timescales. Thus, the characterization of the neural ensemble dynamics in the BF provides further insight into the neural mechanisms, by which brain states are regulated.

## Introduction

Neural population activity has been characterized by measuring several metrics over the past decades. For example, sparseness of cortical population activity has been shown to vary across cell types (Sakata and Harris, 2009; O’Connor et al., 2010; Barth and Poulet, 2012; Harris and Mrsic-Flogel, 2013), and a skewed log-normal distribution of firing rates has been repeatedly shown in cortical and hippocampal neurons (Hirase et al., 2001; Hromadka et al., 2008; Peyrache et al., 2012; Mizuseki and Buzsaki, 2013; Buzsaki and Mizuseki, 2014), imposing biophysical constraints on neural population activity. Correlated spiking is also well documented (Averbeck et al., 2006; Cohen and Kohn, 2011; Doiron et al., 2016) although the functions and underlying mechanisms of such correlated firing are still to be determined (Renart et al., 2010; Litwin-Kumar and Doiron, 2012; Middleton et al., 2012; Mochol et al., 2015; Doiron et al., 2016). Cortical neurons also show rhythmic firing on multiple timescales (Buzsaki and Draguhn, 2004; Schroeder and Lakatos, 2009) and such a rhythmic firing has been implicated in a communication strategy among cortical areas (Buzsaki, 2010; Fries, 2015). However, it is still unclear to what extent such firing regimes can be generalized to other brain areas. Although intensive efforts have been made recently to uncover the structural and functional principles of cortical circuit organization (Kasthuri et al., 2015; Markram et al., 2015; Hawrylycz et al., 2016), it is also important to elucidate any fundamental difference in neural ensemble dynamics between the cortex and other subcortical regions.

The basal forebrain (BF) is a crucial subcortical complex (Mesulam et al., 1983; Woolf, 1991; Zaborszky and Duque, 2003; Zaborszky et al., 2012; Lin et al., 2015; Ballinger et al., 2016). While it consists of multiple “cholinergic” nuclei (Mesulam et al., 1983; Woolf, 1991), BF cells are heterogeneous in terms of molecular, morphological, and electrophysiological properties (Zaborszky and Duque, 2003; Lee et al., 2005; Zaborszky et al., 2012; McKenna et al., 2013; Lin et al., 2015; Xu et al., 2015; Ballinger et al., 2016). While neurodegeneration in the BF is associated with Alzheimer’s disease (Whitehouse et al., 1982; Ballinger et al., 2016), the BF has long been implicated in cortical desynchronization, plasticity, arousal, attention, learning and memory (Everitt and Robbins, 1997; Kilgard and Merzenich, 1998; Zaborszky and Duque, 2003; Weinberger, 2004; Hasselmo and Sarter, 2011; Sakata, 2016). Manipulations of BF neurons modulate cortical and behavioral states in a cell type-specific manner (Kalmbach et al., 2012; Pinto et al., 2013; Eggermann et al., 2014; Anaclet et al., 2015; Kim et al., 2015; Xu et al., 2015; Zant et al., 2016), consistent with the notion that the BF causally regulates arousal. Recent studies have also uncovered links to other cognitive domains, such as decision making and outcome expectations (Lin and Nicolelis, 2008; Hangya et al., 2015; Lin et al., 2015). Thus, much information has been accumulated about the BF, but little is known about how BF neurons act as a population to regulate brain states. Following pioneering works (Lin et al., 2006; Tingley et al., 2015), Dan and colleagues recently investigated cell type-specific activity during both spontaneous and task-related behavior (Harrison et al., 2016). However, BF population activity at high temporal resolution still remains largely unexplored, and in particular it has not been systematically compared with that of cortical population activity.

In the present study, we performed *in vivo* electrophysiological recording in both the mouse BF and auditory cortex (AC), which is a well-characterized cortical area with respect to the structure of neural population activity (Sakata and Harris, 2009, 2012; Harris and Mrsic-Flogel, 2013; Kayser et al., 2015; Sakata, 2016), and to which the anatomical and functional relation of the BF has been investigated (Kilgard and Merzenich, 1998; Weinberger, 2004; Froemke et al., 2007; Chavez and Zaborszky, 2016; Nelson and Mooney, 2016). Comparing the structure of neural spontaneous activity between the BF and AC, here we report that the temporal coordination of AC population activity is highly structured whereas BF populations show less coordination. Our results highlight the importance of comparisons of neural population activity in different brain regions to determine biophysical constraints on the timescale of population activity.

## Materials and Methods

### Animals

A total of 32 transgenic mice expressing channelrhodopsin2 (ChR2) in either cholinergic (22 males, 8 females; ChAT-IRES-Cre::Ai32) (ChAT-IRES-Cre, JAX006410; Ai32, JAX012569) or parvalbumin (2 females; PV-IRES-Cre::Ai32) (PV-IRES-Cre, JAX008069) positive neurons were used in this study. In PV-IRES-Cre::Ai32 mice, only AC recording (*n* = 2) was included in the present study. Experiments were performed in accordance with the United Kingdom Animals (Scientific Procedures) Act of 1986 Home Office regulations and approved by the Home Office (PPL 70/8883).

### *In Vivo* Electrophysiology

We carried out a total of 48 *in vivo* electrophysiological recordings under urethane anesthesia (*n*_anesthetized_ = 27) or unanesthetized head-fixed condition (*n*_unanesthetized_ = 21).

#### Surgical Procedures for Experiments under Anesthesia

Animals were anesthetized with 1.5 g/kg urethane. Lidocaine (2%, 0.1–0.3 mg) was also administered subcutaneously at the site of incision. Two bone screws were fixed in the skull, one in the frontal region (AP +3 mm, ML +2 mm from bregma) used as an electrode for cortical electroencephalograms (EEGs) and one on the cerebellum as a ground and a reference. A craniotomy on the left hemisphere (AP +1 to -1 mm, ML +1 to +3 mm from bregma) was performed to access the BF. For the auditory cortical recording, another craniotomy on the left hemisphere (AP -2 to -4 mm, ML 4–4.5 mm from bregma) was performed. Body temperature was maintained at 37°C with a feedback temperature controller (40–90–8C, FHC).

#### Surgical Procedures for Experiments in Unanesthetized Head-Fixed Condition

Animals were anesthetized with isoflurane (1–1.5%). Lidocaine (2%, 0.1–0.3 mg) was also administered subcutaneously at the site of incision. To provide analgesia after the surgery, Carprofen (Rimadyl, 5 mg/kg) was administered intraperitoneously. A head-post was attached on the skull by implanting two frontal bone screws (AP +3 mm, ML 2 mm from bregma) one of them used for EEG recording. Another two screws were implanted on the cerebellum, one of them used as a ground and a reference. Then, a pair of nuts was attached with dental cement as a head-post. After the head-post surgery the animals were left to recover for at least 5 days. During the acclimation period, the animals were placed in a head-fixed apparatus (SR-8N-S, Narishige), with holding the head-post securely and placing the animal into an acrylic tube. This procedure was continued for at least 5 days, during which the duration of head-fixed was gradually extended from 15 to 60 min. During this period, the animals were also exposed to the sound stimulation in the same manner as the actual electrophysiological recording (see below). A day after this acclimation period, the animals were anesthetized with isofluorane and a craniotomy to access the BF was performed. In some experiments, we carried out a second craniotomy to expose the primary AC in order to carry out a simultaneous BF and AC recording. In the following day, the animals were placed in the head-fixed condition to carry out the unanesthetized electrophysiological recording.

#### Electrophysiological Recordings

Recording procedures are described elsewhere (McAlinden et al., 2015; Sakata, 2016; Scharf et al., 2016). Briefly, all electrophysiological recordings were performed in a single-walled acoustic chamber (MAC-3, IAC Acoustics) with the interior covered by 3 inches of acoustic absorption foam. After the surgical procedures described above, a 32 channel silicon optrode (A1 × 32–Poly3-10 mm–50–177-A32OA or A1 × 32–Poly2-10 mm–50s–177-A32OA, NeuroNexus Technologies) was inserted slowly (2–5 μm/sec) with a motorized manipulator (DMA-1511, Narishige) into the BF (4.0 – 5.0 mm from the cortical surface) at different rostro-caudal and medio-lateral locations. A second 32 channel silicon probe (A1 × 32–Poly2-10 mm–50s–177-A32, NeuroNexus Technologies) was inserted using a manual micromanipulator (SM-25A, Narishige) for AC recordings (800 – 1000 μm from the cortical surface). Both probes were inserted perpendicularly with respect to the cortical surface. The location of the electrode in AC was assessed by evaluating the local field potential (LFP) and multiunit activities (MUA) in response to white noise stimulation, which was generated digitally (sampling rate 97.7 kHz, TDT, Tucker-Davis Technologies) and delivered in free-field through a calibrated electrostatic loud-speaker (ES1) located ∼15 cm in front of the animal.

Broadband signals were amplified (HST/32V-G20 and PBX3, Plexon or RHD2132, Intan Technologies, LLF) relative to a screw anchored in the cerebellar bone and were digitized at 20 kHz (PXI, National Instruments, or RHD2132 and RHD2000, Intan Technologies, LLC). Optical stimulation (see below) was applied during the probe penetration in order to identify cholinergic neurons in the BF. The recording session was initiated > 30 min after the probe was inserted to its target depth, to allow for signal stabilization. A typical recording session consisted of a baseline recording of at least 5 min of spontaneous activity, followed by an optical stimulation protocol and then another baseline of spontaneous activity. During some of recordings, we also played acoustic stimuli, but the results during sound presentations are not reported in the present study.

### Optogenetic Experiments

Detailed procedures are described in previous studies (McAlinden et al., 2015; Scharf et al., 2016). Briefly, blue light (450 or 470 nm, PlexBright, Plexon) was delivered through a fiber optic on the silicon probe. The light output at tip of the probe was measured with a constant long (>1 s) light pulse before probe insertion and was 59.8 ± 17.8 mW/mm^2^ (mean ± SD). Because the first spike latency of BF cholinergic neurons is known to be varied and slow (Unal et al., 2012), we applied optical stimulation for 50 or 100 ms at 2 Hz rate with up to 200 repetitions. However, to reduce the confounding effect of heating (McAlinden et al., 2013; Stujenske et al., 2015; Scharf et al., 2016) and indirect activation, we assessed neural activity only with 50 ms stimulations.

### Histology

For verification of silicon probe tracks, the rear of probes was painted with DiI (∼10% in ethanol, D282, Molecular Probes) before probe insertion. After electrophysiological experiments, animals were perfused transcardially with physiological saline followed by 4% paraformaldehyde/0.1 M phosphate buffer, pH 7.4. After an overnight post-fixation in the same fixative and cryoprotection with 30% sucrose in phosphate buffered saline (PBS), brains were cut into 100 μm coronal sections with a sliding microtome (SM2010R, Leica) and placed in PBS. The sections were mounted on gelatin-coated slides and cover-slipped with antifade solutions and the area of the electrode was photographed using a fluorescence microscope. Based on a pattern of ChR2-EYFP expression, we assessed whether the silicon probe was located in the BF. For a further assessment, sections were also Nissl stained (1% w/v Cresyl Violet plus 1% v/v Glacial Acetic Acid in dH_2_O) to determine sub-nuclei of the BF.

To localize cholinergic neurons in the BF, immunohistochemistry was also performed with some sections. They were incubated with a blocking solution (10% normal donkey serum, NDS, in 0.5% Triton X in PBS, PBST) for 1 h at room temperature followed by incubating primary antibodies (anti-ChAT, 1:200, AB114P, Millipore) in 3% NDS in PBST at 4°C overnight. After washing, sections were incubated with secondary antibodies (donkey anti-boat Alexa Fluor 568, 1:1000, A11057, Life Science Technologies) for 2 h at room temperature. After washing, sections were mounted on gelatin-coated slides and cover-slipped with antifade solution.

### Data Analysis

Data analysis was performed offline using MATLAB (Mathworks) or freely available software. To extract local field potentials (LFPs), a lowpass filter (<100 Hz) was applied and signals were downsampled to 1 kHz. To compute power spectral density, Chronux Toolbox^1^ was used. For spike sorting, the Klusta package (Rossant et al., 2016) was used. This spike sorting process consisted of a semi-automatic process with automatic spike detection and clustering followed by manual clustering, which can reduce spike sorting errors: (1) commission error where spikes belonging to different neurons are clustered together, for example due to synchronous spiking by nearby neurons, and (2) omission errors where not all spikes emitted by a single neuron are grouped together, such as burst firing (Harris et al., 2000; Rossant et al., 2016). After spike sorting, the quality of single units was further assessed by measuring isolation distance (Schmitzer-Torbert et al., 2005). The inclusion criteria for single units in the present study were ≥50 isolation distance and ≥0.1 Hz spontaneous firing. It might be argued that ≥50 isolation distance is apparently too conservative as ≥20 isolation distance has been often set. However, because we used all 32 channels for spike sorting and isolation distance increases depending on the number of features used, we set the inclusion criteria as ≥50 isolation distance.

#### Cell Type Classifications

For BF cells, the optogenetic tagging method was applied (see above). The statistical significance of spike counts in a 50 ms time window during optical stimulations was assessed and compared with those in a pre-stimulus time window (50 ms) by performing Bonferroni corrected rank sum test. A *p*-value of less than 0.05 was recognized as being statistically significant, and significant cells were then categorized as modulated cells. Modulated cells with a significant increase in spike counts were categorized as cholinergic neurons. In the present study, only one neuron showed significant reduction in spike counts, and was categorized as a non-cholinergic neuron within the non-modulated neuron group.

For AC cells, a conventional classification approach was used based on average spike waveforms (Sakata and Harris, 2009, 2012; Sakata, 2016). Briefly, trough to peak time and spike width at 20% of depth were computed from the averaged spike waveforms of each single unit. A threshold of 0.4 ms for trough to peak time and 0.3 ms spike width at 20% of depth was used to classify narrow spiking (NS) or broad spiking (BS) cells.

To estimate the distance of recorded neurons (**Figure 6**), the channel showing the maximum trough to peak amplitude was recognized as the putative somatic position of recorded neurons. Then the distance was determined by the silicon probe design. This estimate was based on observations in previous literature (Henze et al., 2000), in which the amplitude of extracellular spike waveforms becomes the largest at the perisomatic area.

#### Firing Parameter Estimation

Spontaneous firing rate was estimated by counting the total number of spikes during the spontaneous period across single units. To assess a temporal pattern of spiking activity, a proportion of inter-spike intervals (ISIs) with a particular duration *T* (e.g., ≤10 ms) (called ISI_≤__T_) was computed. For a control for this analysis, we took the estimated spontaneous firing rate for each cell to generate a Poisson spike train to compute the same index.

#### Spike Count Correlations

To compute spike count correlations during spontaneous activity, we took the following approach: for neuron *i*, the number of spikes at time *t* was counted as *n*_i_(*t*) in each bin (bin size = 1 ms) convoluted by a Gaussian kernel of standard deviation *T* (*T* = 100 ms). Then spike count correlations between the activity of neuron *i n*_i_(*t*; *T*) and neuron *j n*_j_(*t*; *T*) were computed

$$r_{ij}(T)=\frac{Cov\left(n_{i},n_{j}\right)}{\sqrt{Cov\left(n_{i},n_{i}\right)Cov\left(n_{j},n_{j}\right)}}$$

where *Cov*(*n*_i_, *n*_j_) is the covariance between the activity of the two neurons. We used a MATLAB function, *corrcoef*.

Because neuronal activity is non-stationary and thus can co-vary on a wide range of time-scales, making the evaluation of temporal spike coordination challenging (Grün, 2009), we synthetized surrogate data which maintains a specified mean firing rate for each neuron and specified population rate distribution by using the raster marginals model (Okun et al., 2012). First, the original spike train was binned at 1 ms. Then a binary matrix (0, no spike; 1, spike) was constructed with one column for each time bin and one row for each neuron. To synthetize surrogate data, random 2-by-2 submatrices were repeatedly chosen with each row and column of the submatrix containing a 0 and 1. The positions of 0s and 1s were exchanged in the submatrix, which leaves the summed values of each row and column identical. Compared with a shuffling method (Grün, 2009; Renart et al., 2010) which can preserve the temporal dynamics of population rate of the original data depending on the jittering window size, the raster marginal model discards the temporal structure of the spike trains, with a specified mean firing rate for each single unit and specified population rate distribution.

#### Spike-LFP Phase Analysis

Detailed procedures are described elsewhere (Kayser et al., 2015). Briefly, LFP signal during the spontaneous period was extracted for each single unit. To avoid spurious estimates of spike entrainment to LFPs at high frequency bands, LFP signals were taken from spatially distinct channels that did not contain any spike signals from a given single unit. A Kaiser finite impulse response filter (sharp transition bandwidth of 1 Hz, pass-band ripple of 0.01 dB and stop-band attenuation of 50 dB with forward and backward filtering using MATLAB ‘filtfilt’ function) was used to derive band-limited signals in different frequency bands. In the present study, we assessed the following bands: [2–3], [4–5], [6–8], [8–10], [10–15], [15–20], [20–25], [25–30], [30–40], [40–50], [60–70], [80–90], and [100–110] Hz. The instantaneous phase of each band was estimated from the Hilbert transform and spike phase was computed. To quantify the relationship between spikes and LFP phase, we calculated the percentage of spikes elicited in each phase bin. The rate modulation was defined as the percentage in the preferred bin (the bin with maximal percentage) minus that in the opposite bin (the bin 180° apart). Rayleigh’s test for non-uniformity of circular data was performed to assess the statistical significance (*p* < 0.01) of the non-uniformity of the spike-LFP phase distribution using CircStats Toolbox.

Out of all BF neurons, neurons which showed significant modulation at any frequency band were recognized as rhythmic BF cells. Of these, BF neurons which showed significant modulation at [2–3], [4–5], or [6–8] Hz were recognized as “slow rhythmic BF cells.” In **Figure 7**, spike count correlations were computed in slow rhythmic cell pairs, slow vs. non-slow rhythmic cell pairs, and non-slow rhythmic cell pairs, as described above. In addition, correlations were computed using Gaussian kernels of different standard deviations (5, 10, 25, 50, 75, 100, 150, 200 ms).

### Statistical Analysis

Data were presented as mean ± SEM unless stated. All confirmatory analyses were conducted using MATLAB: Student’s *t*-test was carried out in **Figures 3D,E**. One-way ANOVA with post-hoc Tukey’s honest significant difference (HSD) test was carried out in **Figures 4A,B,D**, **5A**, **7**. Two-way ANOVA with *post hoc* HSD test was carried out in **Figures 4A–C**, **5C,E**. Due to the highly skewed distribution of burstiness (**Figures 4D–J**), Kruskal–Wallis one-way ANOVA with *post hoc* Bonferroni corrected Wilcoxon rank sum test (**Figures 4D,K**) and Wilcoxon rank sum test (**Figures 4E–J**) were carried out. Chi-square goodness-of-fit test was carried out to compare two distributions in **Figures 6B,D**. To estimate effect size, Hedges’ *g* was computed using Measures of Effect Size Toolbox.

## Results

### Database

**FIGURE 1:**
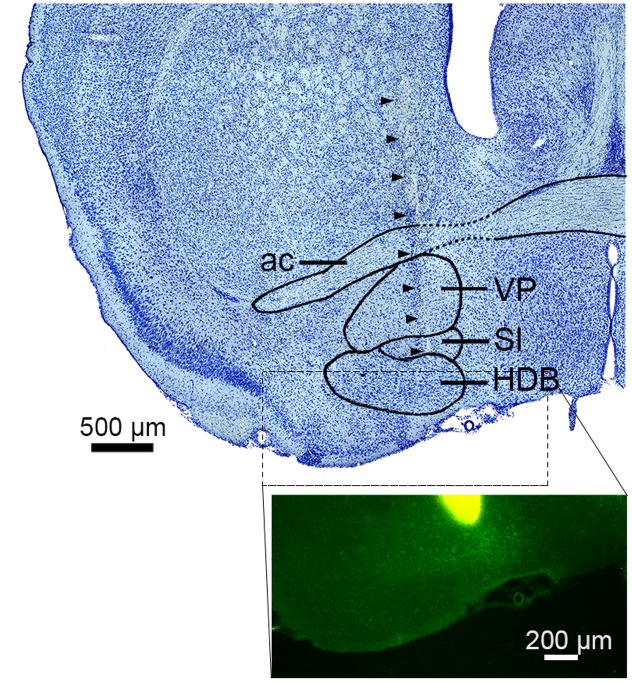
Electrode track in the basal forebrain. **(top)** Nissl-stained section showing the sub-nuclei of the basal forebrain (BF). Arrowheads indicate the track of a silicon probe. ac, anterior commissure; VP, ventral pallidum; SI, substantia innominate; HDB, nucleus of the horizontal limb of the diagonal band. **(bottom)** The tip of a silicon probe was located within the BF (HDB or SI) based on DiI staining. Green signals indicate ChR2-EYFP.

**FIGURE 2:**
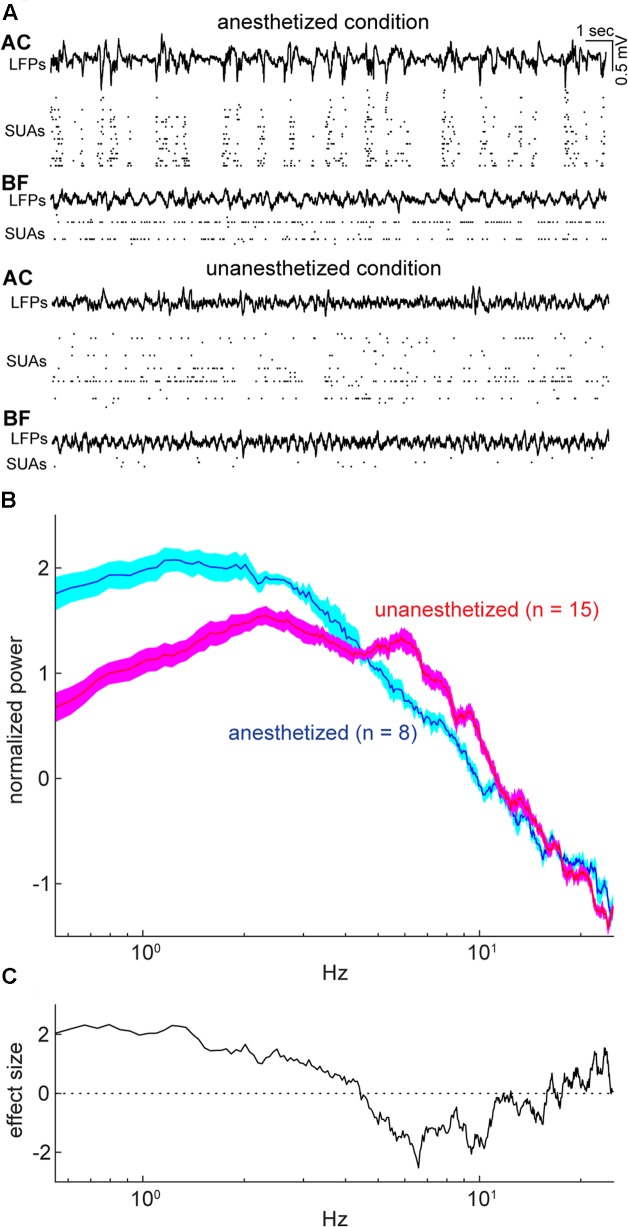
Example simultaneous recording and cortical states during the period of spontaneous activity under anesthetized and unanesthetized conditions. **(A)** Simultaneous recording from the auditory cortex (AC) and basal forebrain (BF) under anesthetized (top) and unanesthetized conditions (bottom), with displaying local field potentials (LFPs) and single-unit activities (SUAs) in both areas. **(B)** Normalized (*Z* scored) power spectral density from spontaneous LFPs in the AC for each experimental condition (*N*_anesthetized_ = 8; *N*_unanesthetized_ = 15). Error indicates SEM. **(C)** Effect size of difference in power spectral density in **(B)**. As expected, the unanesthetized condition was characterized by smaller delta (≤4 Hz) and larger theta (6–10 Hz) components compared to those in the anesthetized condition, indicating that two experimental conditions represent different brain states.

To compare population activity between the AC and BF, we performed *in vivo* electrophysiological experiments in both urethane anesthetized and head-restrained unanesthetized mice. Of 48 recordings from 32 animals, we monitored population activity from the AC in 23 experiments (*n*_anesthetized_ = 8; *n*_unanesthetized_ = 15) and from the BF in 44 experiments (*n*_anesthetized_ = 27; *n*_unanesthetized_ = 17). Electrodes were positioned throughout the BF nuclei, including the basal nucleus of Meynert, nucleus of the horizontal limb of the diagonal band, substantia innominata, extended amygdala, and ventral pallidum (**Figure 1**). Of 44 experiments, eight BF experiments (*n*_anesthetized_ = 5; *n*_unanesthetized_ = 3) were excluded due to the miss-positioning of the electrode following histological assessment. Of the remaining, 15 experiments (*n*_anesthetized_ = 5; *n*_unanesthetized_ = 10) were simultaneous recording of both the BF and AC (**Figure 2A**). Power spectral density of cortical field potentials differed between the anesthetized and unanesthetized conditions (**Figures 2B,C**), indicating that both experimental conditions represent different brain states. Under urethane anesthesia, we observed UP and DOWN states in the mouse AC (**Figure 2A**) as previously reported in the AC and other cortical areas (Steriade et al., 1993; Steriade, 2001; Luczak et al., 2007; Curto et al., 2009; Sakata and Harris, 2009, 2012; Reyes-Puerta et al., 2016; Sakata, 2016). Although population activity could be assessed only during UP states to compare with that during a desynchronized state (Renart et al., 2010; Sakata and Harris, 2012; Sakata, 2016), in the present study we treated the anesthetized state as a single condition.

### Cell Type Classification

We isolated > 300 single units in each area: a total of 357 BF cells (*n*_anesthetized_ = 285; *n*_unanesthetized_ = 72) and 353 AC cells (*n*_anesthetized_ = 130; *n*_unanesthetized_ = 223) were isolated (see Materials and Methods).

**FIGURE 3:**
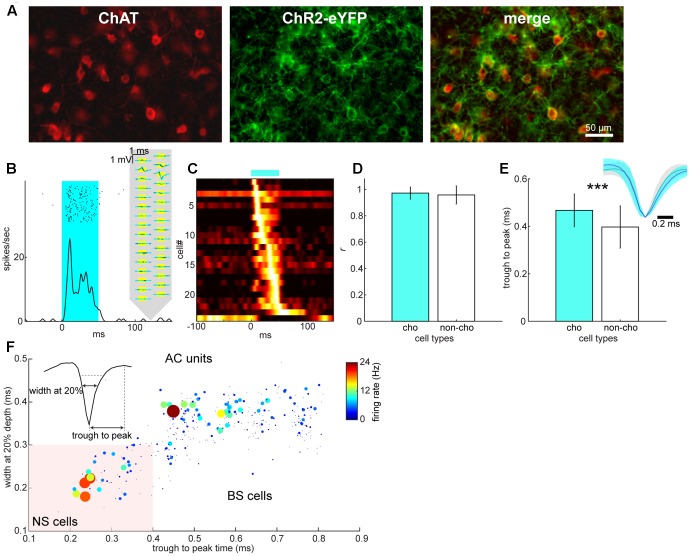
Cell type classification in the BF and AC. **(A)** Expression of ChR2-EYFP in choline acetyltransferase (ChAT)-positive neurons of the BF. **(B)** An example of optical evoked responses in the BF. Spike raster and peri-stimulus time histogram (PSTH) show responses to an optical stimulation (shaded in cyan). Spontaneous (black) and optically evoked (cyan) average spike waveforms are also shown on the schematics of silicon probe. **(C)** A summary of normalized optical evoked responses. Each PSTH was normalized by the peak spike rate and color-coded. Of 24 significantly modulated neurons, one neuron showed suppression although this neuron was categorized as a non-cholinergic neuron. **(D)** Assessment of spike waveform distortion by measuring Pearson’s correlation coefficient with in the population of cholinergic (cho) and non-cholinergic (non-cho) neurons. No significant difference in correlation coefficient between cell types was detected (*t*_330_ = 0.93, *p* = 0.35, Student’s *t*-test). **(E)** Comparison of trough to peak time of average spike waveforms between cholinergic and non-cholinergic neurons (*t*_355_ = 3.72, *p* = 0.00023). Inset: normalized average spike waveforms for cholinergic (blue) and non-cholinergic neurons (black). **(F)** Cell classification of broad-spiking (BS) and narrow-spiking (NS, shaded area) cells based on average spike waveforms. Average spontaneous firing rate is also color-coded with linearly scaled dot size.

We further classified cell types: in the BF, we adopted an optogenetic tagging approach in which channelrhodopsin-2 (ChR2) was expressed in cholinergic neurons (**Figure 3A**) and spikes in ChR2-positive neurons were elicited by optical stimulation during electrophysiological recordings (**Figure 3B**). Optical stimulation significantly modulated 24 out of 357 isolated BF neurons (**Figure 3C**). Of these modulated cells, only one cell showed suppression. Therefore, we classified optically excited cells as cholinergic neurons (*n* = 23) and other cells, including the suppressed cell, as non-cholinergic neurons (*n* = 334). We assessed the distortion of average spike waveforms between optically induced spikes and spontaneous ones in both cholinergic and non-cholinergic neurons by computing Pearson’s correlation coefficient. We did not find any significant difference between cholinergic and non-cholinergic neurons (*p* = 0.35, *t*-test) (**Figure 3D**). However, we found that the spike width (trough to peak time) of cholinergic neurons significantly differs from that of non-cholinergic neurons (*p* < 0.0005, *t*-test) (**Figure 3E**), suggesting physiologically different cell classes. In the AC, we classified neurons into two types based on their spike waveforms (**Figure 3F**). This resulted in 300 broad-spiking (BS) and 53 narrow-spiking (NS) cells, many of which may be parvalbumin-positive interneurons (Madisen et al., 2012).

### Similarities and Differences in Spontaneous Firing Rate and Inter-spike Intervals between BF and AC Neurons

**FIGURE 4:**
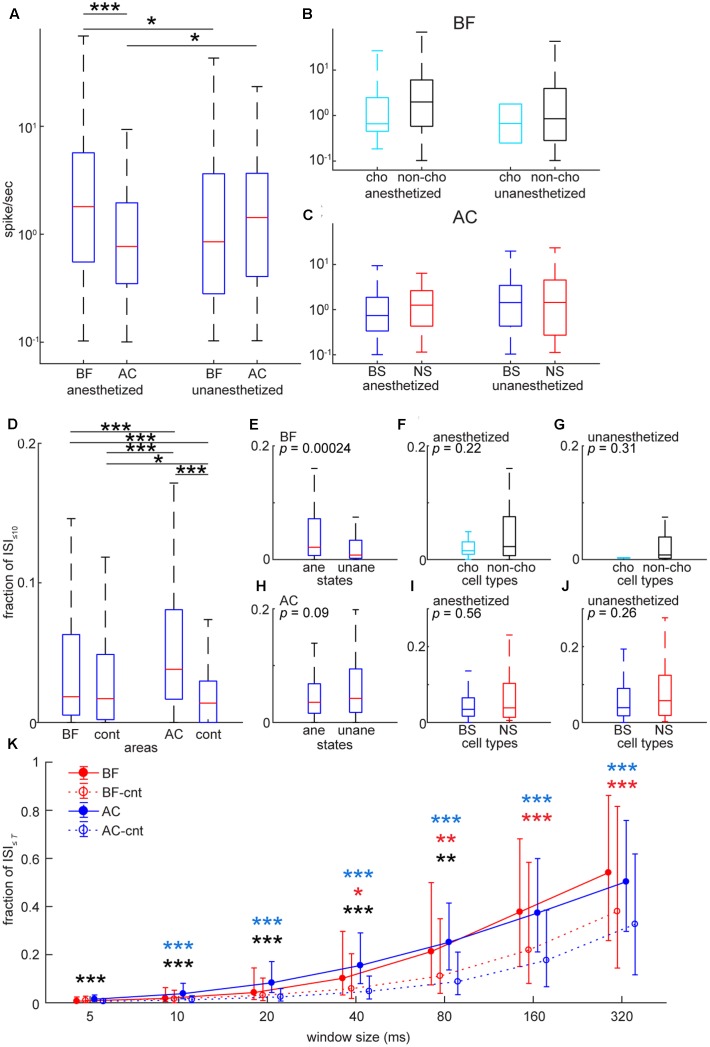
Comparison of spontaneous firing rate and inter-spike intervals in the BF and AC at the single cell level. **(A)** Comparison of average spontaneous firing rate between the BF and AC in log scale. The effect of brain area was significant (*F*_1,706_ = 6.31, *p* = 0.012) whereas the effect between anesthetized and non-anesthetized conditions was not significant (*F*_1,706_ = 0.045, *p* = 0.83). The interaction of brain area and state was significant (*F*_1,706_ = 15.1, *p* = 1.08e–04). ^∗∗∗^*p* < 0.0001; ^∗^*p* < 0.05 [*post hoc* honest significant difference (HSD) test]. **(B)** Comparisons of average spontaneous firing rate between cholinergic (cho) and non-cholinergic (non-cho) neurons in the BF. The effect of cell type (*F*_1,353_ = 1.15, *p* = 0.28) or state (*F*_1,353_ = 0.56, *p* = 0.45) was not significant. No significant interaction of cell type and state was observed (*F*_1,353_ = 0.029, *p* = 0.86). **(C)** Comparisons of average spontaneous firing rate between BS and NS cells in the AC. The effect of cell type (*F*_1,349_ = 0.22, *p* = 0.63) or state (*F*_1,349_ = 2.45, *p* = 0.11) was not significant. No significant interaction of cell type and state was observed (*F*_1,349_ = 0.78, *p* = 0.37). **(D)** Comparison of ISI_≤10_ (a proportion of ≤ 10 ms inter-spike intervals) between the BF and AC. As a control, Poisson spike trains with the same spike rate distribution were included for statistical assessment. The effect of cell group was significant (*X*^2^_3,1416_ = 123, *p* = 1.46e–26, Kruskal–Wallis one-way ANOVA). ^∗^*p* < 0.005, ^∗∗∗^*p* < 0.0001 (*post hoc* Bonferroni corrected Wilcoxon rank sum test). **(E–J)** Comparisons of ISI_≤10_ in the BF **(E–G)** and AC **(H–J)** in different cell types and states. *P*-values were derived from rank sum test. **(K)** Dependency of window size *T* on ISI_≤_*_T_* of BF and AC cells. Median is indicated with quartiles across cell groups. In control groups (BF-cnt and AC-cnt), Poisson spike trains were generated based on mean firing rate. For confirmatory analysis, Kruskal–Wallis one-way ANOVA was performed for each time window *T* (5 ms, *X*^2^_3,1416_ = 76.3, *p* = 1.89e–16; 10 ms, *X*^2^_3,1416_ = 76.3, *p* = 1.85e–16; 20 ms, *X*^2^_3,1416_ = 122, *p* = 2.02e–26; 40 ms, *X*^2^_3,1416_ = 150, *p* = 2.27e–32; 80 ms, *X*^2^_3,1416_ = 147, *p* = 9.08e–32; 160 ms, *X*^2^_3,1416_ = 132, *p* = 1.59e–28; 320 ms, *X*^2^_3,1416_ = 106, *p* = 7.86e–23). ^∗^*p* < 0.05, ^∗∗^*p* < 0.005, ^∗∗∗^*p* < 0.0005 (*post hoc* Bonferroni corrected Wilcoxon rank sum test) (*black*, BF vs. AC; *red*, BF vs. BF-cnt; *blue*, AC vs. AC-cnt).

To assess basic firing properties of BF and AC populations at the single unit level, we first compared spontaneous firing rate (**Figure 4**) between two areas. As shown in **Figure 4A**, the distribution of firing rate was skewed in both areas. To statistically compare mean firing rates in log scale between two areas under two conditions (anesthetized and unanesthetized), a two-way ANOVA was carried out: although the effect of condition was not significant (*F*_1,706_ = 0.05, *p* = 0.83), the effect of brain area was significant (*F*_1,706_ = 6.31, *p* = 0.012) and there was a significant interaction of condition and area (*F*_1,706_ = 15.16, *p* = 0.0001). More specifically, mean firing rate in the BF was higher than that in the AC [*p* < 0.0001, *post hoc* Tukey’s honest significant difference (HSD) test] under anesthesia (**Figure 4A**). Both areas showed changes in firing rate depending on conditions (*p* < 0.05), but in opposite ways. We also examined the effect of cell-type and condition on mean firing rate and their interaction in each area (**Figures 4B,C**). No significant effect (BF: *F*_1,353_ = 1.16, *p*_cell-type_ = 0.28; *F*_1,353_ = 0.56, *p*_condition_ = 0.45) (AC: *F*_1,349_ = 0.22, *p*_cell-type_ = 0.63; *F*_1,349_ = 2.45, *p*_condition_ = 0.11) nor interaction (BF: *F*_1,353_ = 0.03, *p* = 0.86) (AC: *F*_1,349_ = 0.79; *p* = 0.37) was found. Effect size between cholinergic and non-cholinergic neuron firing rates was 0.47 and 0.31 in anesthetized and unanesthetized conditions, respectively. Thus, although no significant cell type specificity was found, the skew distribution of mean firing rate was common in both areas and experimental conditions.

Next, to investigate a temporal structure of firing at the level of individual neurons and its dependency of timescales, we firstly computed the proportion of ≤10 ms inter-spike intervals (ISI_≤10_) (**Figures 4D–J**). AC cells showed significantly higher ISI_≤10_ compared to BF cells (*p* < 0.0005, Kruskal–Wallis test with *post hoc* rank sum test) (**Figure 4D**). To check whether this result can be explained by a Poisson model, we generated a randomized spike train with the same mean firing rate, and then computed ISI_≤10_. There was a significant difference in the median of ISI_≤10_ between real and artificially generated spike trains in the AC (*p* < 0.0005), but not in the BF (*p* = 0.364), suggesting temporally structured firing in AC neurons and a random nature of BF cell firing in a short time scale. Intriguingly, this trend was not held at longer time scales (≥40 ms) (**Figure 4K**). Experimental conditions (anesthetized or unanesthetized) significantly affected this assessment in the BF (*p* < 0.0005, rank sum test) (**Figure 4E**), but not in the AC (*p* = 0.09) (**Figure 4H**). No statistically significant differences between cell types were observed (**Figure 4F**, *p* = 0.225; **Figure 4G**, *p* = 0.311; **Figure 4I**, *p* = 0.563; **Figure 4J**, *p* = 0.261). In sum, although no cell type specificity was found, AC neurons showed temporally organized firing even at short time scales compared to BF neurons at the single cell level.

### Differences in Spike Count Correlations between BF and AC

**FIGURE 5:**
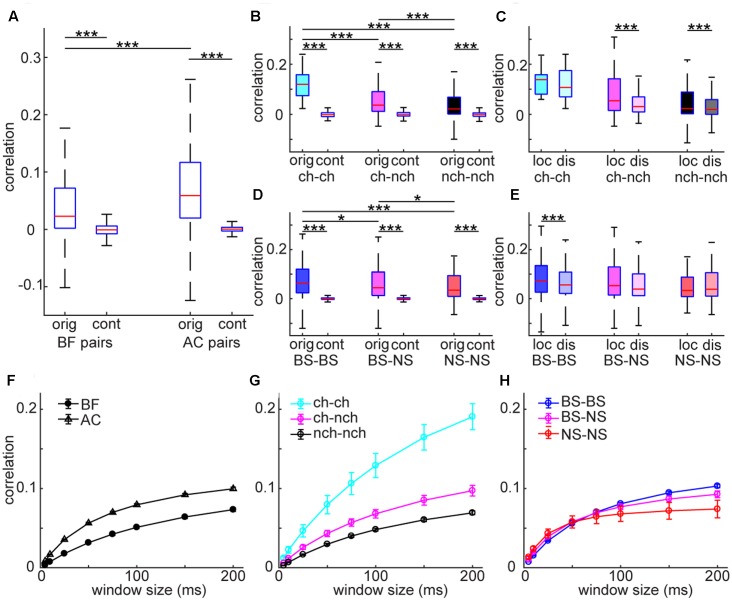
Spike count noise correlations in BF and AC neuron pairs. **(A)** Comparison of correlations between BF and AC pairs. As a control (cont), surrogate data was generated by using the raster marginals model. The effect of pair was highly significant (*F*_3,74455_ = 1148, *p* < 0.00001, one-way ANOVA). ^∗∗∗^*p* < 0.00001 (*post hoc* HSD test). orig, original correlations; cont, control. **(B,D)** Comparisons of correlations across different types of cell pairs in the BF **(B)** and AC **(D)**, with control. The effect of pair type was significant in both the BF (*F*_5,30761_ = 1628, *p* < 0.00001), and AC (*F*_5,43686_ = 5323, *p* < 0.00001). Cholinergic cell pairs showed higher correlations compared to other pairs (*p* < 0.05). ^∗∗∗^*p* < 0.0001 (*post hoc* HSD test within pair types). ch, cholinergic neurons; nch, non-cholinergic neurons. **(C,E)** Comparisons of correlation between local (≤150 μm) and distal (>150 μm) pairs across different types of cell pairs in the BF **(C)** and AC **(E)**. In the BF **(C)**, the effect of pair type was significant (*F*_2,2791_ = 16.6, *p* = 6.56e–08, two-way ANOVA) whereas the effect of distance was not (*F*_1,2791_ = 3.68, *p* = 0.054). A significant interaction of pair type and distance was observed (*F*_2,2791_ = 4.52, *p* = 0.010). In the AC, no effect of pair type (*F*_2,3966_ = 1.34, *p* = 0.25) or distance (*F*_1,3966_ = 1.16, *p* = 0.27) was detected whereas a significant interaction of pair type and distance was observed (*F*_2,3966_ = 3.03, *p* = 0.048). ^∗∗∗^*p* < 0.0001 (*post hoc* HSD test within pair types). **(F–H)** Dependency of window size on spike count correlations. **(F)** Spike count correlations of BF and AC cell pairs across window sizes. **(G)** Spike count correlations of different cell pairs in the BF across window sizes. **(H)** Spike count correlations of different cell pairs in the AC across window sizes.

Analysis at the single cell level suggests differences in the temporal structure of firing between the AC and BF. To investigate the structure of neural firing at the population level, we first quantified temporal correlation between spike trains and asked whether there is any cell type specificity in co-firing. To this end, we computed spike count correlations across recorded neurons in each area (**Figure 5**). AC neurons showed higher correlations compared to BF neurons (*F*_3,74455_ = 11486, *p* < 0.0001, two-way ANOVA with *post hoc* HSD test) whereas both populations showed significantly higher correlations than surrogate data, generated by the raster marginals model (Okun et al., 2012) (*p* < 0.0001) (**Figure 5A**). In the BF, optogenetically identified cholinergic cell pairs showed higher correlations than other pairs (*F*_5,30761_ = 1628, *p* < 0.0001) (**Figure 5B**). Although the number of simultaneously recorded cholinergic cell pairs was very limited in the unanesthetized condition (*n* = 1), this tendency was held regardless of window size (**Figures 5F–H**). Effect size between cholinergic pairs and other pairs (vs. cho–non-cho pairs, 0.64; vs. non-cho–non-cho pairs, 0.91) was also larger than that between other pairs (0.22). In the AC, although we found statistically significant effect of pair types (*F*_5,43685_ = 5323, *p* < 0.0001) (**Figure 5D**), effect size was small (BS-BS vs. BS-NS, 0.040; BS-BS vs. NS-NS, 0.14; BS-NS vs. NS-NS, 0.084). We also assessed an interaction between pair types and distance by separating each pair into two groups depending on estimated distance (local pairs, ≤150 μm; distal pairs, >150 μm) based on the depth profile of average spike waveforms. We found a significant interaction of pair types and distance in both the BF (*F*_2,2791_ = 4.52, *p* < 0.05, two-way ANOVA) (**Figure 5C**) and AC (*F*_2,3966_ = 3.03, *p* < 0.05) (**Figure 5E**), with significant decrease in spike count correlations in some of distal pairs. In summary, spike trains of AC populations showed higher temporal correlation than those of BF populations. Within the BF, cholinergic cell pairs showed higher correlations than non-cholinergic cell pairs.

### Differences in Spike-Field Entrainment between BF and AC

**FIGURE 6:**
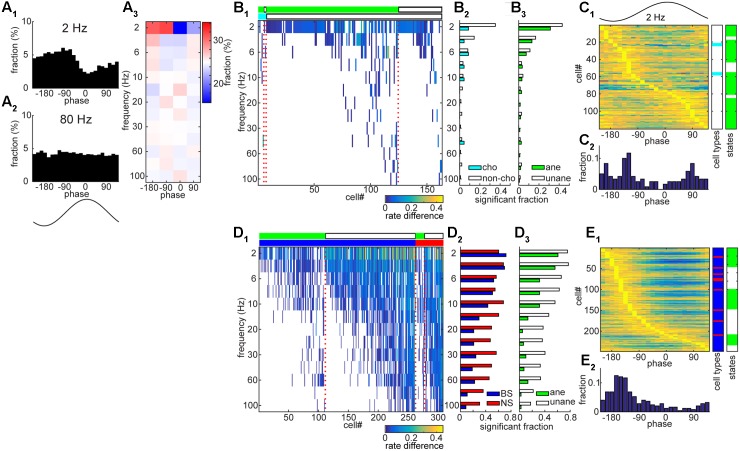
Entrainment of spontaneous spike trains to rhythmic LPFs in the BF and AC. **(A)** An example of BF cell with showing rate modulation by slow (2 Hz) rhythmic LFPs. In **(A_1_)** and **(A_2_)**, the fraction of spikes is shown as a function of LFP phase at a particular frequency band. In **(A_3_)**, phase bins were divided into four phases and the fraction of spikes was color-coded. **(B)** A summary of rate modulations across frequency bands in the BF. In **(B_1_)**, rate difference was defined as the difference in the fraction of spikes at between the peak phase and 180° opposite phase. Rate difference was color-coded only at frequencies with significant modulation (*p* < 0.01, Rayleigh’s test). Different experimental conditions (light green, anesthetized; white, unanesthetized) and cell types (cyan, cholinergic cells; white, non-cholinergic cells) are shown in the top bars, respectively. In **(B_2_,B_3_)** the fraction of cells which showed significant modulations was shown. No significant difference in the distribution was detected (**B_2_**, *p* = 0.98; **B_3_**, *p* = 0.99, chi-square goodness-of-fit test). **(C)** Phase modulations of BF cells at 2 Hz. The fraction normalized by the peak value was shown in **(C_1_)** with information about cell types and experimental conditions. In **(C_2_)** the distribution of peak phases was shown. **(D)** A summary of rate modulations across frequency bands in the AC. In **(D_1_)**, rate difference was color-coded. Different experimental conditions (light green, anesthetized; white, unanesthetized) and cell types (blue, BS cells; red, NS cells) are shown in the top bars, respectively. In **(D_2_,D_3_)** the fraction of cells which showed significant modulations was shown. A significant difference in the distribution was detected (**D_2_**, *p* = 1.2e – 23; **D_3_**, *p* = 6.0e – 63). **(E)** Phase modulations of AC cells at 2 Hz.

To further investigate the temporal coordination between spiking and net synaptic activity in both the BF and AC, we examined to what extent spikes entrained to ongoing field potentials across different frequency bands (2 – 100 Hz) (**Figure 6**). A handful of BF neurons showed significant entrainment to slow components of LFPs (**Figures 6A–C**), suggesting rhythmic firing within the BF. As shown in **Figure 6A**, this example BF neuron clearly showed rhythmic firing at 2 Hz, but not at 80 Hz. Of 357 BF neurons, 163 cells (45.6%) showed significant rhythmic modulations at any of analyzed frequency bands, but mainly at slow (≤6 Hz) frequencies (*n* = 140, 39.2%). We did not find significant differences in the distribution of modulated cells across frequencies, either among cell types (*p* = 0.98, chi-square goodness-of-fit test) or conditions (anesthetized vs. unanesthetized conditions) (*p* = 0.99). In addition, preferred phases at 2 Hz also varied across cells (**Figure 6C**). In contrast to BF populations, AC neurons showed highly structured firing (**Figures 6D,E**), showing entrainment in 87.2% of cells (308/353) at any of the frequency bands analyzed (**Figure 6D_1_**). Notably there is a clear cell type specificity (*p* < 0.0001, chi-square goodness-of-fit test), with a higher fraction of NS cells showing significant modulations at higher frequencies (>10 Hz) (**Figure 6D_2_**). A larger fraction of AC cells showed entrainment under the unanesthetized condition (*p* < 0.0001) (**Figure 6D_3_**). AC neurons showed a unimodal distribution of peak phase at 2 Hz (**Figure 6E**). Thus, the AC neuronal population shows temporally coordinated activities on multiple timescales whereas a smaller subset of BF neurons shows spike-field entrainment especially at slow (≤6 Hz) frequencies.

### Higher Spike Count Correlations of Slow Rhythmic BF Cells

**FIGURE 7:**
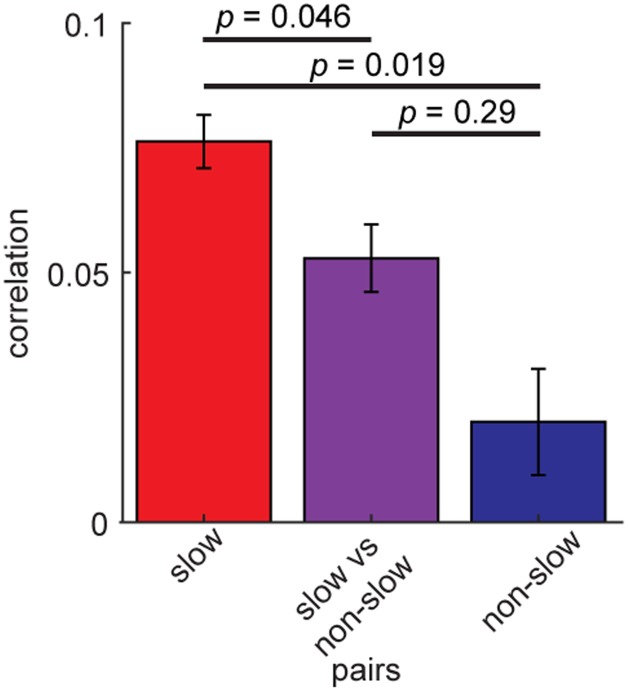
Higher spike count correlations of slow rhythmic BF cells. Mean spike count correlations of slow (≤6 Hz) rhythmic BF cells (*n* = 140) and other rhythmic BF cells (*n* = 23). The effect of pair type was significant (*F*_2,801_ = 5.74, *p* = 0.0033, one-way ANOVA). *P*-values of *post hoc* HSD test are shown. Errors indicate SEM.

Finally, we examined whether coordinated spiking of the rhythmic BF neurons differs depending on their rhythmic modulations. To this end, we re-assessed spike count correlations between the rhythmic BF cells (*n* = 163) by dividing into two groups, slow (≤6 Hz) (*n* = 140) and non-slow rhythmic cells (*n* = 23) (**Figure 7**). Slow rhythmic BF cell pairs showed significantly higher correlations compared to other cell pairs in a 100 ms time window (*F*_2,801_ = 5.74, *p* = 0.0033, two-way ANOVA). This was also the case in different time windows (75–200 ms). Thus, firing of slow rhythmic BF neurons was more correlated than that of other rhythmic BF neuron pairs.

## Discussion

The distribution of spontaneous firing rate was skewed in both the AC and BF. However, BF populations showed firing statistics distinct from those of AC populations, with BF cells firing densely under anesthesia and showing less temporally structured firing at a short time scale (≤20 ms), which is largely explained by a Poisson model. At the population level, BF cells showed lower spike count correlations, with the exception of cholinergic cell pairs, and less spike-field entrainment to LFPs, compared to AC cells. Overall, the fundamental difference in the structure of population activity between the AC and BF is their operational timescales. Given the modulatory nature of BF populations at a relatively slow timescale, less temporally coordinated firing may be appropriate to modulate downstream populations. Rather, cortical circuits should be recognized as a specialized neural circuit allowing signal transmission on multiple timescales.

The present study builds on previous studies in the BF (Lin et al., 2006; Tingley et al., 2015) by using an optogenetic tagging approach along with cortical ensemble recording. Similar optogenetic approaches have been recently used in the BF *in vivo* (Hangya et al., 2015; Xu et al., 2015). Although we identified a statistically significant difference in spike waveforms between cholinergic and non-cholinergic cells, it should be noted that there are several limitations in electrophysiologically identifying true cholinergic cell type *in vivo*. Given that neurons are interconnected irrespective of cell type within the BF (Zaborszky and Duque, 2000, 2003; Zaborszky et al., 2012; Yang et al., 2014; Xu et al., 2015), it is possible that optically evoked responses may be observed even in non-cholinergic neurons. To avoid this confounding effect, one may take neurons with rapid onset responses as cholinergic neurons (Hangya et al., 2015; Xu et al., 2015). However, it is also known that some of cholinergic neurons indeed show delayed spiking (Unal et al., 2012), suggesting that a short light pulse (e.g., less than 100 ms) may not be able to elicit spikes in cholinergic neurons. There is, therefore, a trade-off between false positive and negative, depending on the time window being assessed and the light pulse duration being used. Although we cannot exclude the possibility that there may have been false positive and negative in the present study, our observations are consistent with previous studies: firstly, the proportion of optogenetically identified cholinergic neurons (23/357 = 6.4%) is consistent with a stereological estimate (∼6.2%) (Gritti et al., 2006). Secondly, we found that the spike width of cholinergic neurons significantly differs from that of non-cholinergic neurons, consistent with brain slice experiments (Unal et al., 2012). On the other hand, we are also aware that the proportion of cholinergic neurons is varied across reports (2–14%) (Hangya et al., 2015; Xu et al., 2015). This discrepancy is probably due to light pulses (duration and power), ChR2 expression (virus or transgenic), optrode designs (wire or silicon probe) and sampled nuclei. To address this issue further, cell type specificity in the BF will need to be confirmed by other means in future, such as an imaging technology with high temporal resolution.

Basal forebrain populations showed lower spike count correlations, suggesting that BF cells fire more independently compared to cortical cells. Interestingly, optogenetically identified cholinergic neuron pairs show higher correlations. Given the small number of cholinergic neurons, this may be functionally reasonable in order to have an impact on the downstream as a population. On the other hand, inferring the underlying mechanisms of such higher correlations is not trivial: a recent comprehensive anatomical study indicates that the inputs to different cell types are similar (Do et al., 2016). Therefore, a subtle quantitative difference in long-range inputs and/or local connections may give rise to this cell type-specificity. In addition to further anatomical investigations, physiological and computational explorations will also be essential in addressing this issue, given the complexity of the biophysical mechanisms of correlated spiking (Renart et al., 2010; Mochol et al., 2015; Doiron et al., 2016). This is also the case for the mechanisms of higher correlations among slow rhythmic BF cells.

In the AC, we employed a conventional approach to classify cell classes based on spike waveforms (Sakata and Harris, 2009, 2012; Sakata, 2016). While it is likely that many of NS cells were parvalbumin-positive cells, BS cells likely consisted of heterogeneous cell classes, including not just excitatory cells, but also other inhibitory cell classes. This technical difficulty may have introduced discrepancies between the current results and previous reports (Sakata and Harris, 2012; Sakata, 2016). However, we confirmed a significant proportion of NS cells entrained to higher frequency bands, especially gamma bands, consistent with previous reports (Tremblay et al., 2016).

In the present study, we performed experiments under both anesthetized and unanesthetized conditions. Effects of condition were complex: Although we find a clear difference in frequency contents of cortical LFPs (**Figure 2**), neuronal activity at the single cell level provides a complex picture: spontaneous firing rate in unanesthetized AC is higher compared with that in anesthetized AC, consistent with previous reports (Sakata and Harris, 2009, 2012), whereas BF neuronal activity decreased in the unanesthetized condition. This BF activity may reflect diversity of state-dependent spontaneous firing across cell types (Zaborszky and Duque, 2003; Lee et al., 2005; Lin et al., 2006; Xu et al., 2015). In the BF, no statistically significant difference in rhythmic firing was found between conditions (**Figure 6**) whereas the AC showed a clear difference, that is, more AC cells showed rhythmic firing across a range of frequencies in the unanesthetized condition. These results support the notion that AC populations transmit signals on multiple timescales especially in the unanesthetized condition, and AC activity is more coordinated compared with BF population activity.

Why is AC population activity more orchestrated compared to BF population activity? Their anatomical organization may provide insights; although cortical neurons are highly heterogeneous, the cytoarchitecture in the cortex is highly modular (Szentagothai, 1983). On the other hand, the BF lacks such a cytoarchitecture. Accordingly, input and output organizations are also fundamentally different: in the cortex, incoming fibers are spatially organized and downstream targets also differ depending on cortical layers and cell types (Szentagothai, 1983; Linden and Schreiner, 2003; Douglas and Martin, 2004; Harris and Shepherd, 2015). In the BF, however, different cell types are intermingled and the anatomical organization is less modular (Zaborszky et al., 2015). These anatomical differences between both areas may help explain the underlying mechanisms of population activity in both structures. In addition to this, diversity of cortical inhibitory interneurons also plays a crucial role in the organization of cortical population activity (Tremblay et al., 2016). Compared with cortical interneurons, however, little is known about the local functional interactions in the BF *in vivo*, in particular functional roles of a diverse set of inhibitory neurons (Zaborszky and Duque, 2000; Zaborszky et al., 2012; Xu et al., 2015; Do et al., 2016). Therefore, further investigations are essential to mechanically understand the fundamental difference in the organization of population activity between the cortex and BF.

Functionally, our results suggest that the operational timescale seems to be different between the BF and cortex. Although recent studies have appreciated fast actions of BF neurons (Sarter et al., 2009; Pinto et al., 2013; Hangya et al., 2015; Tingley et al., 2015), again the timescale is still slower than that in cortical operations, such as in a gamma range (tens of milliseconds). Our findings may also impose biophysical constraints on BF population activity for brain functions. For example, there is compelling evidence that gamma coherence plays a critical role in neuronal communications (Fries, 2005, 2015). However, this theory may not be applicable for BF populations. Rather our results support the hypothesis that BF populations play a role in enhancing the communication through gamma coherence in the cortex as BF parvalbumin-positive projection cells play a causal role in cortical gamma oscillations (Kim et al., 2015).

Are there any general principles of population activity across the AC and BF? Although the structure of population activity in the AC and BF differs markedly, the skewed distribution of spontaneous firing rate is common across areas and states (Buzsaki and Mizuseki, 2014). Although technical caveats (e.g., sampling bias) have to be taken into account, the normal distribution should not be applied to model population activity in either structure. The difference in firing rate across cortical cell classes is also associated with anatomical and functional constraints as we have proposed previously (Sakata and Harris, 2009; Harris and Mrsic-Flogel, 2013). It may be conceivable that BF cells also show a similar tendency, which may be seen by analyzing different cell types more systematically. Another principle is the effect of correlated spiking on the downstream. Although the time scale differs between the BF and cortex, co-firing seems to have a larger impact onto the downstream. Thus, although much effort has been made recently in systematically elucidating cortical circuits (Kasthuri et al., 2015; Markram et al., 2015; Hawrylycz et al., 2016), comprehensive comparisons of population activity between different brain areas and cell types would provide further insights into the organizational principles of neural circuits.

## Author Contributions

JY and SS designed and conceived experiments. JY, TT, and SS performed electrophysiological and optogenetic experiments. JY performed histological experiments. JY and SS analyzed data. JY and SS wrote the manuscript with input from TT.

## Conflict of Interest Statement

The authors declare that the research was conducted in the absence of any commercial or financial relationships that could be construed as a potential conflict of interest.

## Footnotes

1 http://chronux.org/
